# Establishment and Validation of a Prediction Equation to Estimate Risk of Intraoperative Hypothermia in Patients Receiving General Anesthesia

**DOI:** 10.1038/s41598-017-12997-x

**Published:** 2017-10-24

**Authors:** Jie Yi, Lujing Zhan, Yongjing Lei, Shiyuan Xu, Yongyu Si, Shiyang Li, Zhongyuan Xia, Yisa Shi, Xiaoping Gu, Jianshe Yu, Guohai Xu, Erwei Gu, Yonghao Yu, Yanqing Chen, Hequn Jia, Yinglin Wang, Xiuli Wang, Xiaoqing Chai, Xiaoju Jin, Junping Chen, Meiying Xu, Junyu Xiong, Guonian Wang, Kaizhi Lu, Wenli Yu, Weifu Lei, Zaisheng Qin, Jingguo Xiang, Longyun Li, Min Yao, Yuguang Huang

**Affiliations:** 1 Chinese Academy of Medical Sciences & Peking Union Medical College Hospital, Beijing, China; 2 3M R&D Center, Shanghai, China; 3 The Second People’s Hospital of Wuhu, Wuhu, China; 4 Zhujiang Hospital of Southern Medical University, Guangzhou, China; 5 The Second Affiliated Hospital of Kunming Medical University, Kunming, China; 6 Quanzhou Women’s and Children’s Hospital, Quanzhou, China; 7 Renmin Hospital of Wuhan University, Wuhan, China; 8 Lanzhou University Second Hospital, Lanzhou, China; 9 Nanjing Drum Tower Hospital, The Affiliated Hospital of Nanjing University Medical School, Nanjing, China; 10 The Affiliated Hospital of Inner Mongolia Medical University, Inner Mongolia, China; 11 The Second Affiliated Hospital of Nanchang University, Nanchang, China; 12 The First Affiliated Hospital of Anhui Medical University, Hefei, China; 13 General Hospital of Tianjin Medical University, Tianjin, China; 14 Fujian Provincial Hospital, Fuzhou, China; 15 The Fourth Hospital of Hebei Medical University, Shijiazhuang, China; 16 Haikou People’s Hospital, Haikou, China; 17 The Third Hospital of Hebei Medical University, Shijiazhuang, China; 18 Anhui Provincial Hospital, Hefei, China; 19 The First Affiliated Hospital of Wannan Medical College, Wuhu, China; 20 Ningbo No. 2 Hospital, Ningbo, China; 21 Shanghai Chest Hospital Shanghai Jiaotong University, Shanghai, China; 22 The Second Hospital of Dalian Medical University, Dalian, China; 23 Harbin Medical University Cancer Hospital, Harbin, China; 24 The First Affiliated Hospital of Chongqing Third Military Medical University, Chongqing, China; 25 The First Central Hospital Tianjin, Tianjin, China; 26 Qilu Hospital of Shandong University, Jinan, China; 27 Southern Medical University Nanfang Hospital, Guangzhou, China; 28 The People’s Hospital of Sanya, Sanya, China; 29 Sino-Japanese Friendship Hospital of Jilin University, Changchun, China

## Abstract

Inadvertent intraoperative hypothermia (core temperature <36 °C) is a frequent but preventable complication of general anesthesia. Accurate risk assessment of individual patients may help physicians identify patients at risk for hypothermia and apply preventive approaches, which include active intraoperative warming. This study aimed to develop and validate a risk-prediction model for intraoperative hypothermia. Two independent observational studies in China, the Beijing Regional Survey and the China National Survey, were conducted in 2013 and 2014, respectively, to determine the incidence of hypothermia and its underlying risk factors. In this study, using data from these two studies, we first derived a risk calculation equation, estimating the predictive risk of hypothermia using National Survey data (3132 patients), then validated the equation using the Beijing Regional Survey data (830 patients). Measures of accuracy, discrimination and calibration were calculated in the validation data set. Through validation, this model, named Predictors Score, had sound overall accuracy (Brier Score = 0.211), good discrimination (C-Statistic = 0.759) and excellent calibration (Hosmer-Lemeshow, P = 0.5611). We conclude that the Predictors Score is a valid predictor of the risk of operative hypothermia and can be used in deciding whether intraoperative warming is a cost-effective measure in preventing the hypothermia.

## Introduction

Mild inadvertent intraoperative hypothermia (core temperature<36 °C) is common among patients undergoing major operations under general anesthesia^1^. Hypothermia has been reported in 4% to 72% of cases^1^, and up to 90% in some studies^2,3^. The hypothermia is associated with several major adverse events that increase patients’ operative risks: cardiac complications^4,5^; hemorrhage^6–8^; and infection^9–13^, especially in patients with high-risk factors (American Society of Anesthesiologists grade III, IV). The decrease in body temperature may also lead to altered drug metabolism^14,15^, prolonged time in a post-anesthetic care unit or intensive care unit^16^, and uncomfortable cold for patients. These consequences can affect patients’ satisfaction with the procedure and increase the cost^17,18^.

From the standpoint of patient safety, body temperature is one of the vital signs and must be monitored carefully during the perioperative period. A final core temperature of ≥ 36 °C is widely considered “normothermic,” and it has been incorporated into various clinical guidelines^19^. For example, the Surgical Care Improvement Project suggested a final intraoperative temperature above 36 °C and use of active over-body warming for body temperature below 36 °C^20^. In the United States, according to guidelines^22^, all surgical patients are actively warmed. And active warming is suggested but is uncommon in many other developed Western countries^21^. In developing countries, though, few surgical patients are actively warmed.

In this study, we aimed to develop a readily accessible method for quantifying individual patient’s absolute risk of intraoperative hypothermia. Using two independent data sets from two recent observational studies in China^22,23^, we strove to derive and externally validate a prediction equation to determine the hypothermia risk. We believe this information could help patients be aware of the risk and help physicians identify high-risk patients, make informed treatment decisions, and more closely manage modifiable risk factors.

## Results

### Incidence of Hypothermia

In the Beijing Regional Survey, the incidence of intraoperative hypothermia among the 830 patients was 39.9%. As is standard of care in China, all patients were warmed passively by covering with surgical drapes or cotton blanket; 10.7% of patients also received active warming with space heater, electric blanket, or other devices. Pre-warmed intravenous fluid was infused in 16.9% of patients, and 34.6% were rinsed with pre-warmed fluid.

In the China National Survey, the incidence of intraoperative hypothermia was as high as 44.3%, and the average rates of hypothermia were 17.8%, 36.2%, 42.5% and 44.1% within 1 h, 2 h, 3 h and 4 h, respectively, after induction of anesthesia. As in the Beijing Survey, all patients were warmed passively; a low percentage (14.2%) were also given active warming. Baseline characteristics of derivation and validation cohorts are summarized in Table 1.

**Table 1:** Baseline Characteristics of Derivation and Validation Cohorts.

Variables	Derivation Cohort (n = 3132)	Validation Cohort (n = 830)
**Age**		
mean ± std (yr)	53.51 ± 13.80	50.1 ± 16.9
<=65 n (%)	2502 (79.89)	723 (88.1)
>65 n (%)	630 (20.11)	107 (12.9)
**Gender**, **n (%)**		
Male	1468 (46.87)	500 (60.2)
Female	1664 (53.13)	330 (39.8)
BMI, mean ± std	23.60 ± 3.60	24.7 ± 7.2
**ASA, n (%)**		
1	335 (10.70)	282 (33.8)
2	2431 (77.62)	468 (56.1)
3	358 (11.43)	78 (9.4)
4	8 (0.25)	4 (0.5)
**Type of Surgery, n (%)**		
General Surgery	1303 (41.60)	234 (28.2)
OB/GYN	533 (17.02)	227 (27.4)
Peripheral Vascular Surgery	9 (0.29)	12 (1.5)
Cardiovascular Surgery	458 (14.62)	8 (0.9)
Thoracic Surgery	7 (0.22)	70 (8.4)
Orthopedic Surgery	332 (10.60)	126 (15.2)
Neurosurgery	1 (0.03)	22 (2.7)
Urology	431 (13.76)	72 (8.7)
Plastic Surgery	3 (0.10)	27 (3.3)
Others	55 (1.75)	32 (3.9)
**Magnitude of Surgery, n (%)**		
minor	12 (0.38)	5 (0.6)
intermediate	243 (7.76)	169 (20.4)
major	1235 (39.43)	421 (50.7)
major plus	1642 (52.43)	235 (23.8)
**Invasiveness of surgery, n (%)**		
endoscopic surgery	1597 (50.99)	427 (51.5%)
open surgery	1535 (49.01)	403 (48.5%)
**Mode of Anesthesia, n (%)**		
general	2988 (95.40)	783 (94.3)
general + regional	144 (4.60)	47 (5.7)
Total Anesthesia Time, mean ± std, (h)	2.97 ± 1.49	2.5 ± 1.5
Overall Incidence of Hypothermia (%)	44.30%	39.90%
**Baseline core temperature prior to anesthesia (°C)**		
mean ± std	37.2 ± 0.3	37.1 ± 0.4
**OR ambient temperature (°C)**		
mean ± std	22.9 ± 1.4	23.5 ± 1.7
Patient receiving intraoperative passive warming, n (%)	3132 (100.0)	830 (100.0)
Patient receiving intraoperative active warming, n (%)	443 (14.17)	89 (10.7)
**Blood transfusion**		
**autologous**		
n (%)	75 (2.4)	52 (6.3)
mean ± std (ml)	519.8 ± 372.2	429.9 ± 259.3
median (ml)	447	400
min-max (ml)	0–2250	100–1250
**allogeneic**		
n (%)	317 (10.1)	52 (6.3)
mean ± std (ml)	735.2 ± 500.6	791.4 ± 777.2
median (ml)	600	800
min-max (ml)	50–3540	150–5800
**Perioperative IV fluid**		
**n (%)**		
mean ± std (ml)	1768.3 ± 819.3	1702.5 ± 914.9
patients receiving prewarmed IV fluid, n (%)	649 (20.7)	141 (16.9)
patients receiving unwarmed IV fluid, n (%)	2483 (79.3)	689 (83.0)
**Intraoperative irrigation fluid**		
**n (%)**		
mean ± std (ml)	1879.9 ± 8194.5	650.4 ± 975.4

### Predictor Variables

Several predictor variables were selected based on published literature^1,2,24^ of significant contributors to intraoperative hypothermia, then examined in the two studies separately. Analysis showed that higher BMI (>=25), higher patient baseline core temperature prior to anesthesia, and higher ambient temperature prevented hypothermia. In contrast, major operations, longer duration of anesthesia (>2 h), more intravenous un-warmed fluid infusion (>1000 ml), and open surgery increased the risk of hypothermia. Intraoperative active warming, although not with recommended forced-air warming, reduced the chance of hypothermia (Table 2).

**Table 2:** Multivariable Model and Risk Factors Associated with Intraoperative Hypothermia (OR, 95% Confidence Interval).

**Risk Factors**	**Derivation Cohort (n = 3132)**	**Validation Cohort (n = 830)**
**Age**		
<=65	reference	reference
>65	1.04(0.64–1.69)	0.72(0.42–1.23)
**Gender**		
Male	reference	reference
Female	0.73(0.49–1.09)	0.76(0.53–1.09)
**ASA**		
<3	reference	reference
≥3	0.79(0.42–1.47)	1.01 (0.55–1.87)
**Magnitude of Surgery**		
Minor/Intermediate Surgery	reference	reference
Major & Major + Surgery	1.98(1.25–3.13)	1.93(1.27–2.95)
**Anesthesia**		
General Anesthesia alone	reference	reference
Anesthesia (combined)	1.69(0.71–4.01)	1.84 (0.85–3.99)
**IV fluid replacement (>1000 ml)**		
≤1000 ml	reference	reference
>1000 ml	2.54(1.48–4.37)	2.78 (1.74–4.46)
**Intraoperative irrigation**		
≤500 ml	reference	reference
>500 ml	1.30(1.10–1.54)	0.73 (0.47–1.15)
**Duration of Anesthesia**		
≤2 h	reference	reference
>2 h	2.07(1.37–3.12)	3.284 (1.81–5.95)
**Endoscopic Surgery**		
yes	reference	reference
no	1.36(0.92–1.99)	0.76 (0.53–1.09)
**Patient Warming**		
passive warming	reference	reference
active warming	0.36(0.19–0.66)	0.44 (0.25–0.78)
**Baseline core temperature before anesthesia (C°)**	0.07(0.03–0.14)	0.075 (0.04–0.13)
**BMI**		
> =25	reference	reference
<25	0.42(0.29–0.61)	0.39 (0.28–0.56)
**OR ambient temperature (C°)**	0.87(0.77–0.98)	0.88 (0.79–0.98)

The final regression model was expressed as:

$$\begin{array}{c}{\rm{Predictors}}\,{\rm{Score}}={\rm{Probability}}(P)\,{\rm{of}}\,{\rm{Hypothermia}}\,\times \,100 \% =100 \% \\ \qquad \quad \qquad \quad \quad \,\,\times 1/\{1+\mathrm{EXP}[-(119\,+\,0.201\,\times \,{\rm{Magnitude}}\_{\rm{Surgery}}\,-\,0.1847\\ \qquad \quad \qquad \quad \quad \,\,\times {\rm{Intravenous}}\_{\rm{Fluid}}\,+\,0.5299\times {\rm{Duration}}\_{\rm{Anesthesia}}-0.2269\\ \qquad \quad \qquad \quad \quad \,\,\times {\rm{Mode}}\_{\rm{of}}\_{\rm{Patient}}\_{\rm{Warming}}\,-\,0.306\,\times \,{\rm{BMI}}\,-\,0.1912\\ \qquad \quad \qquad \quad \quad \,\,\times {\rm{OR}}\_{\rm{Ambient}}\_{\rm{Temperature}}-3.1057\,\times \,{\rm{Baseline}}\_{\rm{Core}}\_{\rm{Temperature}})]\},\end{array}$$

where R-squared = 0.15. The Predictors Score is not applicable to patients under 18 and cardiovascular surgeries with extracorporeal circulation as we did not include these patients and types of surgeries in both Beijing Regional and National Hypothermia Survey^22,23^.

### Performance of Regression Model

**Figure 1:**
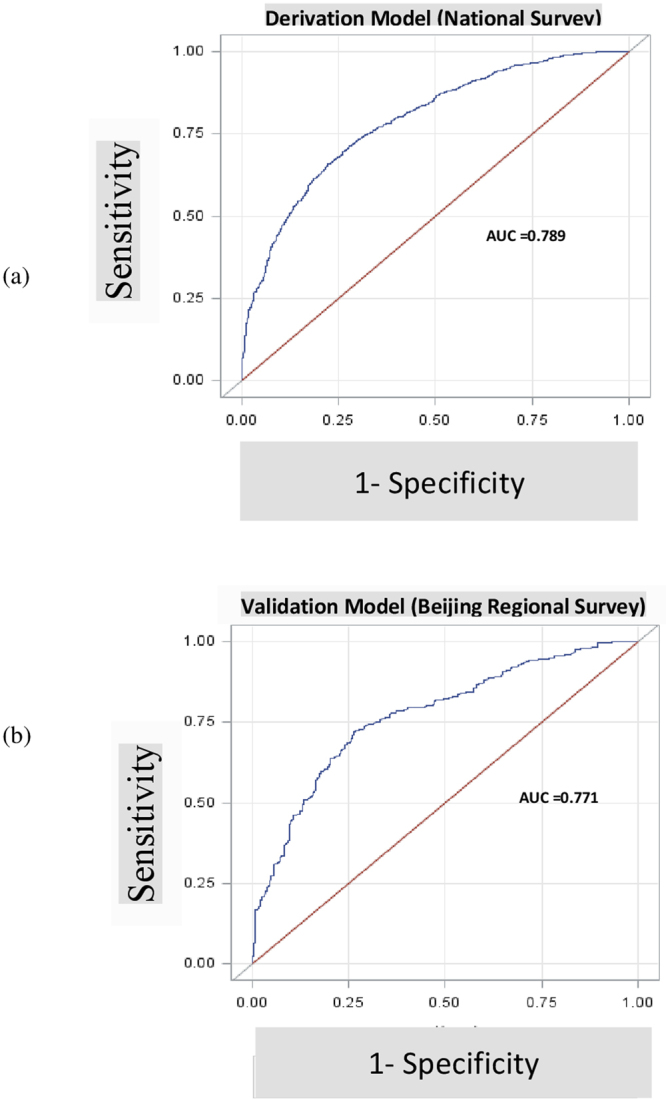
Performance of Derivation Model (**a**) and Validation Model (**b**).

Based on receiver operation curve analysis, C-statistics of derivation and validation sets were 0.789 and 0.771, respectively, indicating good discrimination (Fig. 1). The Hosmer-Lemeshow goodness-of-fit statistic was used to test model reliability in the validation cohort, and P = 0.5611 indicated a good match of predicted risk over observed risk. Brier Score is a measure of calibration defined as the means squared difference between predicted hypothermia and actual hypothermia; Brier Score = 0.21 represents a good accuracy.

### Risk Calculator

**Figure 2:**
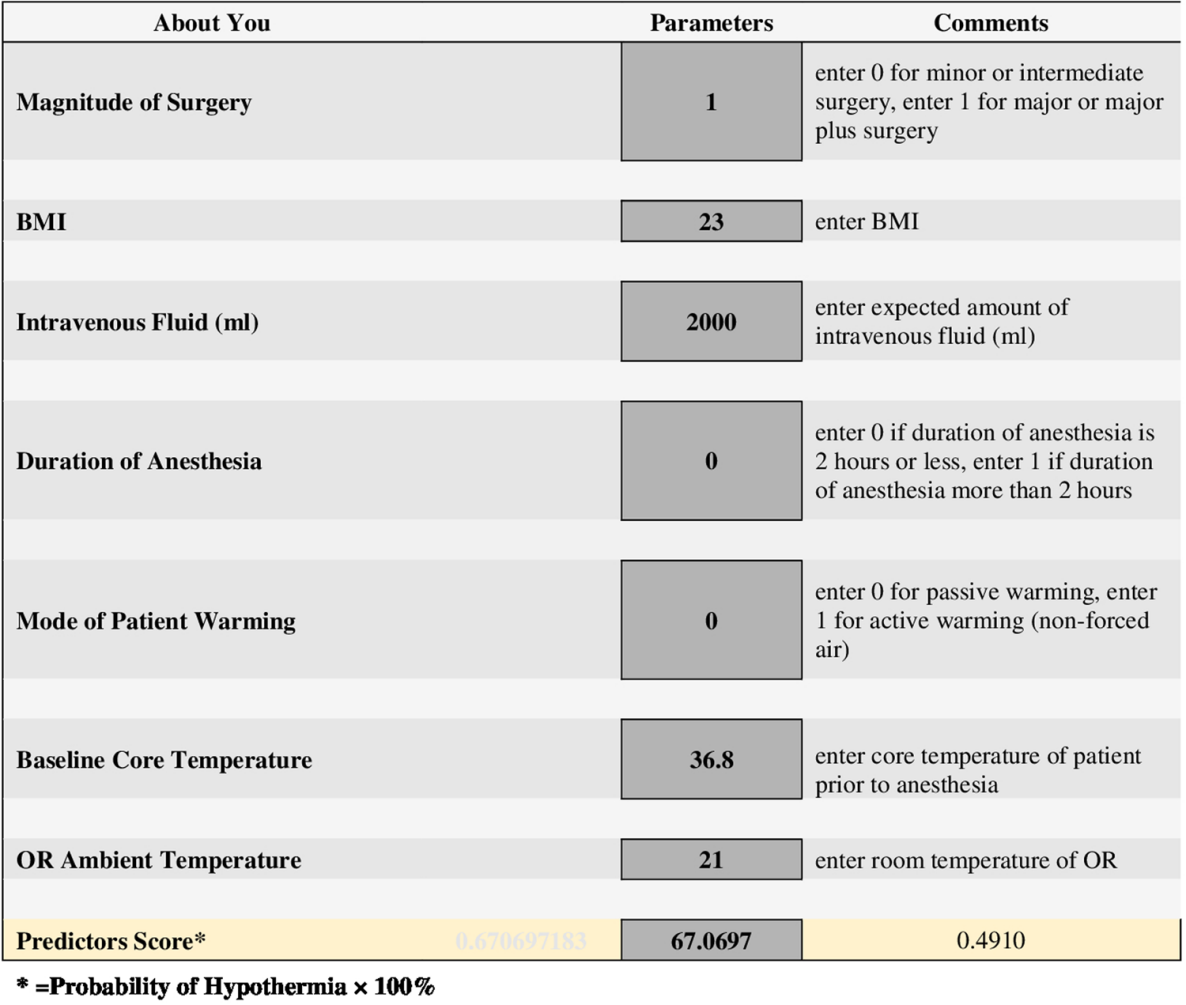
Risk Calculator to Determine Possibility of Intraoperative Hypothermia.

The risk calculator that implements the risk equation from the final model was created with Excel Spreadsheet (the presenting spreadsheet is shown in Fig. 2). We named the calculated probability of hypothermia “Predictors Score,” which implies the quantitative risk of hypothermia. The Predictors Score, which is simply equal to the Probability (P) of Hypothermia × 100% (see also above), represents the predicted risk for each patient of developing intraoperative hypothermia given the baseline information and hypothesized variables before and during the operation.

## Discussion

Intraoperative hypothermia and its adverse consequences are known by medical practitioners throughout the world, but limited data of hypothermia had been available in China. This is one of the reasons that we initiated regional and national studies on intraoperative hypothermia since 2013. It has also been difficult to develop policies regarding hypothermia in a big country such as China, with its geographic, socioeconomic, and cultural diversity and variations in healthcare resources. Thus, we aimed in this study to develop a readily accessible method for quantifying an individual patient’s absolute risk of intraoperative hypothermia.

Towards this end, we developed and then externally validated a hypothermia risk prediction calculator for patients undergoing general anesthesia. The equations are well performed and demonstrated good discrimination with, C statistic values of 0.759 in the validation cohort, a sound overall accuracy (Brier Score) and goodness of fit (Hosmer-Lemeshow). To our knowledge, this is the first such tool to quantify the risk of hypothermia for individual patients.

Perioperative core temperature is affected by multiple factors, including patients’ health and illnesses, the operation, and the anesthesia. These variables are differently weighted as they contribute to hypothermia. In this study, we first screened literature and reviewed our clinical experience, then selected a group of risk factors associated with hypothermia from one data set (national data) and validated it with another data set (Beijing data). Initially, we included all known risk factors associated with hypothermia: age, gender, American Society of Anesthesiologists physical status, magnitude of surgery, BMI, mode of anesthesia, volume of intravenous fluid used, volume of irrigation fluid used, duration of anesthesia, endoscopic/open surgery, patient-warming mode, baseline core temperature, and operating room ambient temperature. However, as with the risk- assessment model generated from the Framingham Heart Study^25^ and in other studies^26^, we revised this full model to a simplified one with fewer but highly weighted risk factors. The final model includes only magnitude of surgery, duration of anesthesia, BMI, baseline core temperature, operating room ambient temperature, volume of intravenous fluid infused, and the mode of patient warming. This list, we believe, is more practicable for health care providers to use. By entering a risk-factor profile, a patient’s risk of hypothermia, which we named Predictors Score, can be generated. Use of the Score should help physicians identify high-risk patients; assess the risk vs benefit, including cost of active perioperative active warming; and help in management of modifiable risk factors. The scores may also help patients beware of the risk of hypothermia.

Although perioperative warming is routine practice in the United States and many other developed countries, it is not often used in most developing countries or in China; amounts and allocation of healthcare resources likely is a determinant in this difference. To follow United States’ guidelines, reliable warming of patients to 37 °C or higher usually requires 30 minutes of pre-warming, two intraoperative forced-air warming covers, and a fluid warmer, all of which add cost to the operation^24^. This expense may not be necessary for many operations, such as small operations performed in a warm operating room. The need for scrutiny in the use of perioperative warming is one of the reasons we initiated regional and national studies on hypothermia in intraoperative patients in China.

There are some limitations in our study. First, both Beijing and National studies did not include patients under 18 years old, so the risk assessment applies only to adult patients. Second, although the risk assessment calculator was generated from the national survey, then validated by the Beijing regional survey, repressiveness may still be an issue; due to lack of technical capability, patients were not selected in a randomized manner in the national survey.

In summary, we developed and validated a regression equation, “Predictors Scores,” for predicting personalized risk of hypothermia in patients undergoing general anesthesia. The potential usefulness of the Score is to help physicians identify patients at risk for hypothermia before commencement of anesthesia, and in deciding whether active perioperative warming is needed. This assessment of risk should aid in making sound clinical decisions and in the rational use of healthcare resources.

## Methods

### Patients and Data Source

Data were derived from two independent observational studies: the Beijing Regional Survey^23^ and the China National Survey for Intraoperative Hypothermia^22^. The studies are parts of the China National Anesthesiology Healthcare Quality Improvement Program. The Beijing Regional Survey is a regional cross-sectional study in the city of Beijing, conducted from June 2013 through December 2013. Eight hundred and thirty eligible patients who underwent various operations under general anesthesia were randomly selected from 24 hospitals in Beijing through a multistage probability sampling^23^.

The China National Survey for Hypothermia and Patient Warming, another observational study, was conducted from November 2014 through August 2015. It is a prospective study with 30-day postoperative follow-up to identify the incidence and clinical outcome of hypothermia. A total of 3132 eligible patients who underwent general anesthesia in 28 hospitals nationwide were enrolled. Data access is available (see data availability statement below).

This is a retrospective observational study based on the data obtained from two previous studies, the Beijing Survey and the National Survey, which were led by Peking Union Medical College Hospital (PUMCH). This study was carried out in accordance with Good Clinical Practice guidelines and regulations. The study protocol was fully approved by Institutional Review Board (IRB) of PUMCH and then informed consent form (ICF) was waived.

### Model Derivation and Validation

Because the Beijing Regional Survey and the China National Survey are independent observational studies for intraoperative hypothermia, we first used the China National Survey cohort to derive a multivariate logistic regression model and identify predictors associated with intraoperative hypothermia and their relative predictive weights (coefficients). We selected and examined these predictor variables based on published literature^1,2,24^. The variables were: age, gender, intraoperative active warming, body mass index (BMI), baseline core temperature prior to anesthesia, ambient temperature of the operation room, magnitude of operation, duration of anesthesia, volume of intravenous un-warmed fluid used, volume of intraoperative irrigation used, and open/endoscopic surgery. The model was then validated with another data set from the Beijing Regional Survey.

### Model Performance

Model performance was evaluated with C statistic, Brier Score and Hosmer-Lemeshow Goodness-of-Fit statistics^27–29^. C statistic, or area under the curve (AUC) of receiver operating characteristics, is a measure of discrimination, with 0.5 corresponding to discrimination that is no better than chance, and 1.0 corresponding to perfect prediction. The Brier Score is a measure of calibration defined as the means squared difference between predicted outcome and actual outcome (occurrence of hypothermia in this study), with the score = 0.0 representing a perfect model performance. Additionally, Hosmer-Lemeshow Goodness-of-Fit Statistics was calculated to evaluate model reliability; a large P value (>0.05) indicates a good match of predicted risk over observed risk. After model validation, a risk calculator based on the model using the National Survey cohort was established. All data manipulation and statistical analysis were performed using SAS 9.4 (SAS Institute, Inc.).

### Data Availability

Our data are available from PUMCH Institutional Data Access/Ethics Committee for researchers only who meet the criteria for access to confidential data. Please submit an application form, obtainable at http://www.pumch.cn to Prof. Jie Chen, Chair of PUMCH Institutional Review Board. Researchers requesting data access may also contact corresponding author Dr. Yuguang Huang. Upon agreement, researchers should sign a data usage contract with PUMCH specifying terms and conditions.
